# A randomized controlled trial of exercise to prevent muscle mass and functional loss in elderly hemodialysis patients: Rationale, study design, and baseline sample

**DOI:** 10.1016/j.conctc.2019.100365

**Published:** 2019-04-19

**Authors:** Khin N. Chan, Yu Chen, Yiming Lit, Payam Massaband, Jenny Kiratli, Ralph Rabkin, Jonathan N. Myers

**Affiliations:** a Cardiology Division, Veterans Affairs Palo Alto Health Care System, United States; b Nephrology Division, Veterans Affairs Palo Alto Health Care System, United States; c Nephrology Division, Stanford University, United States; d Radiology Division, Veterans Affairs Palo Alto Health Care System, United States; e Spinal Cord Injury Center, Veterans Affairs Palo Alto Health Care System, United States; f Cardiology Division, Stanford University, United States

**Keywords:** Home-based exercise, Cardiopulmonary exercise testing, Elderly, Hemodialysis, Randomized clinical trial, Protein signaling

## Abstract

**Background:**

Elderly maintenance hemodialysis (MHD) patients exhibit muscle wasting and impaired physical function. This trial determines whether MHD patients benefit from a 12-week home-based exercise program, protein supplementation, or both.

**Design:**

and Methods: This is a randomized, blinded controlled trial involving 60 elderly MHD patients with impaired exercise capacity and function. Patients are randomized into either a homebased exercise program or normal care over a 12-week period. Measures at baseline include peak VO_2_, strength and body composition as well as cognitive and disease-specific questionnaires. Muscle biopsies are obtained and analyzed for protein signaling, expression of IGF-1, androgen receptors, and myostatin.

**Results:**

At baseline, patient characteristics in the exercise and normal care groups were similar by age, gender and anthropomorphic measures. Peak VO_2_ was impaired (14.7 ± 3.3 ml/kg/min), representing 55 ± 14% of the age-predicted value. Six-minute walk distance was 322 ± 71 m, and the mean 1-min sit to stand test was 18 ± 8 repetitions, representing 69 ± 16% and 55 ± 22% of the age-predicted values, respectively. Indices of muscle function, including upper and lower body and hand grip strength all indicate marked impairment. Quality of life (QoL) using the SF36, the Beeson cognitive test, and KDQOL all suggest marked impairments compared to age-expected reference values for non-MHD patients.

**Conclusions:**

Patients undergoing MHD exhibit markedly reduced physical function and QoL. Thus, there are potentially significant gains to be made through a program of aerobic and resistance exercise. We anticipate this trial will demonstrate that home-based exercise improves cardiopulmonary function, protein signaling and QoL, and increases muscle mass, strength, and body composition.

## 1 Introduction

In chronic kidney disease (CKD) muscle mass and physical function decline as renal failure progresses [1]. These processes are accelerated in elderly dialysis patients since both the uremic environment and aging cause loss of muscle mass and function that together predispose these patients to frailty. This inevitably leads to an increase in fall-related injuries, a decrease in quality of life (QOL) and an increase in morbidity and mortality [2,3]. The mortality rate among CKD patients on maintenance hemodialysis (MHD) increases by 2-fold in those aged 66–74 and approximately 3-fold in those aged 75–79 vs. those <65 years [4,5]. Current recommendations to prevent and manage aging-related adverse events in MHD include provision of adequate nutrient intake and regular exercise [[6], [7], [8], [9], [10]]. Despite recommendations for exercise-based rehabilitation in end-stage renal disease (ESRD) patients, implementation of rehabilitation programs as a standard treatment modality is limited [8,9].

Several studies in patients with ESRD have reported that both intensive and moderate aerobic training improve cardiovascular performance. This may be particularly important for ESRD patients, who have a high prevalence of cardiovascular disease that accounts for nearly half of their deaths [4,5,]. In addition, regular endurance exercise can increase muscle fiber size in ESRD patients to an extent that is similar to that in age-matched reference subjects [[11], [12], [13], [14], [15]]. Most of the exercise studies in patients with ESRD have involved small numbers, lacked randomized controls, were of short duration, and enrolled relatively young subjects in relatively good condition; few data are available regarding elderly ESRD patients [5]. In addition, there is limited information on the cellular mechanisms underlying the salutary effect of exercise in humans with ESRD. Moreover, the precise relationship between muscle wasting, loss of function and poor long-term outcomes in elderly ESRD patients remains to be fully explored. Thus, there is a need for large-scale studies to establish the true benefits of exercise in this population.

Therefore, we have undertaken a randomized proof of concept trial. As a primary outcome, we anticipate that regular exercise will increase cardiopulmonary function measured by peak VO_2_. As secondary outcomes, we expect that exercise therapy will increase muscle mass and improve psychosocial health, strength, balance and anthropometric measures, counteract muscle wasting and reduce cardiac risk factors. In addition, we postulate that the provision of a high calorie leucine-rich protein supplement at the time of acute exercise will enhance exercise-stimulated anabolic signaling. Finally, we address the mechanisms by which regular exercise counteracts muscle wasting in elderly ESRD patients and whether they are explained by changes in local skeletal muscle expression of IGF-1, androgen receptors and myostatin, and by stimulating Akt signaling pathways that promote protein synthesis and inhibit proteolysis.

## 2 Methods

***Overall study design***. The study is a randomized controlled trial comparing 12 weeks of home-based exercise training vs. usual care on exercise capacity, muscle mass and function in patients with CKD who are undergoing MHD.

***Study setting.*** The study is being conducted at the Veterans Affairs Palo Alto Healthcare System (VAPAHCS), an affiliate of Stanford University. The study was approved by the Stanford Panel on Human Subjects. The study is funded by a grant from the VA Rehabilitation Research and Development Service and is registered on ClinicalTrials.gov.

***Participant eligibility.*** The VAPAHCS Nephrology Division, Stanford Transplant Readiness and Anticipatory Care (*TRAC)* clinic, and local dialysis clinics are participating as part of a network-based recruitment strategy. Men and women aged 55–80 years with impaired exercise capacity (peak VO_2_ 10–20 ml/kg/min) who are undergoing MHD for at least three months with an average Kt/V ≥ 1.2 are potentially eligible. Patients with temporary vascular access, uncontrolled diabetes mellitus, active autoimmune disease, malignancy, severe obesity (BMI >35), alcoholism or other recreational drug use, unstable cardiac disease (abnormal exercise test, angina, uncontrolled arrhythmias or myocardial infarction within three months), peripheral vascular disease (claudication with exercise), and those who are medically unstable are excluded from the study. In addition, patients who are currently active (>2 h/week of moderate intensity exercise), or who have received anabolic, catabolic or cytotoxic medications in the past 3 months are excluded from participation.

***Recruitment process.*** Subjects are recruited from the VAPAHCS, Stanford University Medical Center, local Satellite Dialysis Inc. clinics within 30–40 miles of the VAPAHCS, and local nephrologists with travel reimbursement provided. We initially contacted 11 local Satellite Dialysis clinics for in-service presentations regarding the study purpose and recruitment process. In addition, we sent out formal letters and study fliers with a project summary to local nephrologists for potential recruitment. We screened medical charts for eligibility (with clinician oversight), and those who remained eligible after chart review were contacted about the study. Those interested in participation gave written informed consent and authorization to release medical information from their local Satellite Dialysis clinic before being scheduled for study visits.

The study has two major aims, with specific procedures as follows.

## 3 Aim 1

We plan to develop a low-cost aerobic and resistance exercise regimen to counteract the loss of muscle mass and function common in elderly MHD patients that is easily implemented, suitable for the home and to which most subjects will adhere. As a primary outcome, we anticipate that regular exercise will increase cardiopulmonary function measured by peak VO_2_. Peak VO_2_ is an independent predictor of survival in ESRD patients and the gold standard for cardiopulmonary fitness. As secondary outcomes, we expect that exercise therapy will increase muscle mass and improve psychosocial health, strength, balance and anthropometric measures, counteract muscle wasting and reduce cardiac risk factors.

Together, these measures should allow us to determine whether the home-based exercise regimen is effective in counteracting loss of muscle function and mass common in elderly MHD patients, along with reducing cardiovascular risk.

**Aim 1 study procedures:** Table 1 outlines the study measures. Symptom-limited exercise testing is performed using an individualized ramp treadmill protocol or cycle ergometer (depending upon patient stability) to measure maximal oxygen consumption (peak VO_2_) at baseline and 12 weeks. Secondary metrics include anthropometric measurements (mid-arm circumference, abdominal girth, and skinfold thickness), lower and upper body strength, body composition, thigh muscle mass, muscle biopsy for protein signaling, quantitative muscle morphology and gene expression, daily activities, QOL, laboratory measures of cardiovascular risk factors, and nutritional and inflammatory parameters. Strength is measured using leg extension one repetition maximum for lower body strength and chest press one repetition maximum for upper body strength. Maximal isometric strength is determined using a hand-grip dynamometer. Patients perform a 6-min walk test in a corridor, walking from end to end at their own pace. A 60 s sit to stand test is performed to measure balance and muscle power; this includes time to complete five repetitions and number of repetitions completed within 60 s.

**Table 1:** Study measures.

Measurement	Baseline	6 weeks†	12 weeks
- Vitals, weight & physical evaluation	✓		✓
Body Composition & Muscle Area			
- Thigh & Waist Circumference	✓		✓
- MRI or CT scan for thigh volume & composition	✓		✓
- DXA for body composition	✓		✓
Fitness and Functional Assessments			
- Maximal cardiopulmonary exercise test (peak VO_2_)	✓		✓
- Six-minute walk test (6 MWT)	✓		✓
- One-minute sit-to-stand test	✓		✓
Strength Test (1 RM, upper and lower body)			
- Chest press	✓		✓
- Leg extension	✓		✓
- Hand grip	✓		✓
Muscle Biochemistry			
- Muscle biopsies (vastus lateralis)	✓		✓
- Expression of muscle mass regulators	✓		✓
- Muscle morphology	✓		✓
Questionnaires			
- Physical activity questionnaire			
- SF-36 v2 physical (PCS) and mental (MCS) components	✓		✓
- Kidney Disease Quality of Life Form (KDQOL)	✓		✓
- Cognitive test (Beeson cog CRF)	✓		✓
Fasting Bloods			
Blood chemistry, full blood count, lipid panel, others	✓	✓	✓

A whole-body dual X ray densitometry (DXA) scan is performed to assess total body lean mass and percent body fat. Either a CT or MRI is performed on the mid-thigh to determine thigh muscle mass and to calculate percentage of intramuscular fat.

Participants complete a quality of life (SF36V_2_) and kidney disease-specific quality of life survey (KDQOL) to assess perceived well-being in physical, psychological, and social areas of health. In addition, a battery of five cognitive tests is employed to assess cognitive domains including visuospatial scanning, psychomotor speed, executive function, attention, verbal learning, memory and verbal cognitive flexibility. These measures are repeated week 12. Fasting blood samples are collected in the dialysis clinic through vascular access at baseline, week 6 and week 12 to determine changes in nutritional parameters, lipid panels, C-reactive protein, inflammatory cytokines, and complete blood count.

***Exercise Intervention***. Participants randomized to the exercise group undergo a 12-week individualized exercise program, prescribed by an exercise physiologist. The individualized exercise programs involve a combination of supervised and home-based monitored exercise. Exercising participants receive four supervised exercise sessions over the initial two weeks at the hospital followed by home-based exercise sessions throughout the study. Participants are given hand-held weights, Thera-bands in accordance with their capabilities, and cycle ergometers for home use, along with a heart rate monitor. Participants are instructed to strictly adhere to their exercise prescriptions and encouraged to perform a combination of continuous aerobic activities and resistance exercise for a minimum of 45 min per day. Daily activity logs are used to record activities, intensity, duration, and heart rate (HR). Exercise intensity is targeted to achieve 70–80% of HR reserve and 12–14 on the Borg perceived exertion scale. In-clinic exercise sessions are repeated every two weeks to ensure stability and compliance, and to modify the exercise prescription as appropriate. Participants also receive a weekly follow up phone call by study coordinators to monitor their clinical status and activities.

Participants not randomized to the exercise intervention receive continued standard clinical care throughout the study period.

## 4 Aim 2

Aim 2 involves: a) elucidating the mechanisms whereby a program of exercise-based rehabilitation counteracts muscle wasting in elderly MHD patients; and b) determining whether the mTOR (mammalian target of rapamycin) anabolic signaling response to exercise or a high-calorie leucine-rich protein load is intact in elderly MHD patients and whether the response to exercise is enhanced by providing this supplement at the time of exercise.

**Aim 2 Study Procedures.** A muscle biopsy is performed at baseline and at the end of week 12. The muscle biopsy is designed to quantify protein signaling, quantitative muscle morphology and gene expression. At the baseline visit, the vastus lateralis muscle of the non-dominant leg is biopsied under local anesthesia and sterile conditions with a Bergtrom needle. At the post-study visit (week 12), the vastus lateralis muscle of both legs is biopsied 100–120 min after acutely exercising the dominant leg to fatigue using repeated leg extension. Participants are administered either a high-calorie leucine rich protein or a placebo drink at the beginning and immediately after acute exercise on the morning of the biopsy to determine the effects of leucine supplementation on protein signaling following acute and chronic exercise. The nutritional supplement is double-blinded and randomly assigned through a centralized computer process to either placebo or whey protein and prepared by a VA pharmacist.

***Randomization procedure and sample size.*** Following completion of the baseline visit, patients are randomized using a 1:1 ratio to either the exercise or normal care group. Sample size estimates are based on our primary outcome (comparison of the change in peak VO_2_ for patients randomized to exercise vs. normal care). The target change in peak VO_2_ of 17% was based on a summary of previous studies and the fact that a change of this magnitude has been associated with favorable functional and QOL outcomes in patients with CKD. For sample size estimates, we assumed that there would be no change in the control group. To detect a difference in group means of 17% at 80% power and assuming a type I error of 5%, the target sample size is 30 subjects per group. This number allows for a 20% dropout rate, resulting sample sizes of 23 subjects per group.

**Fig. 1:**
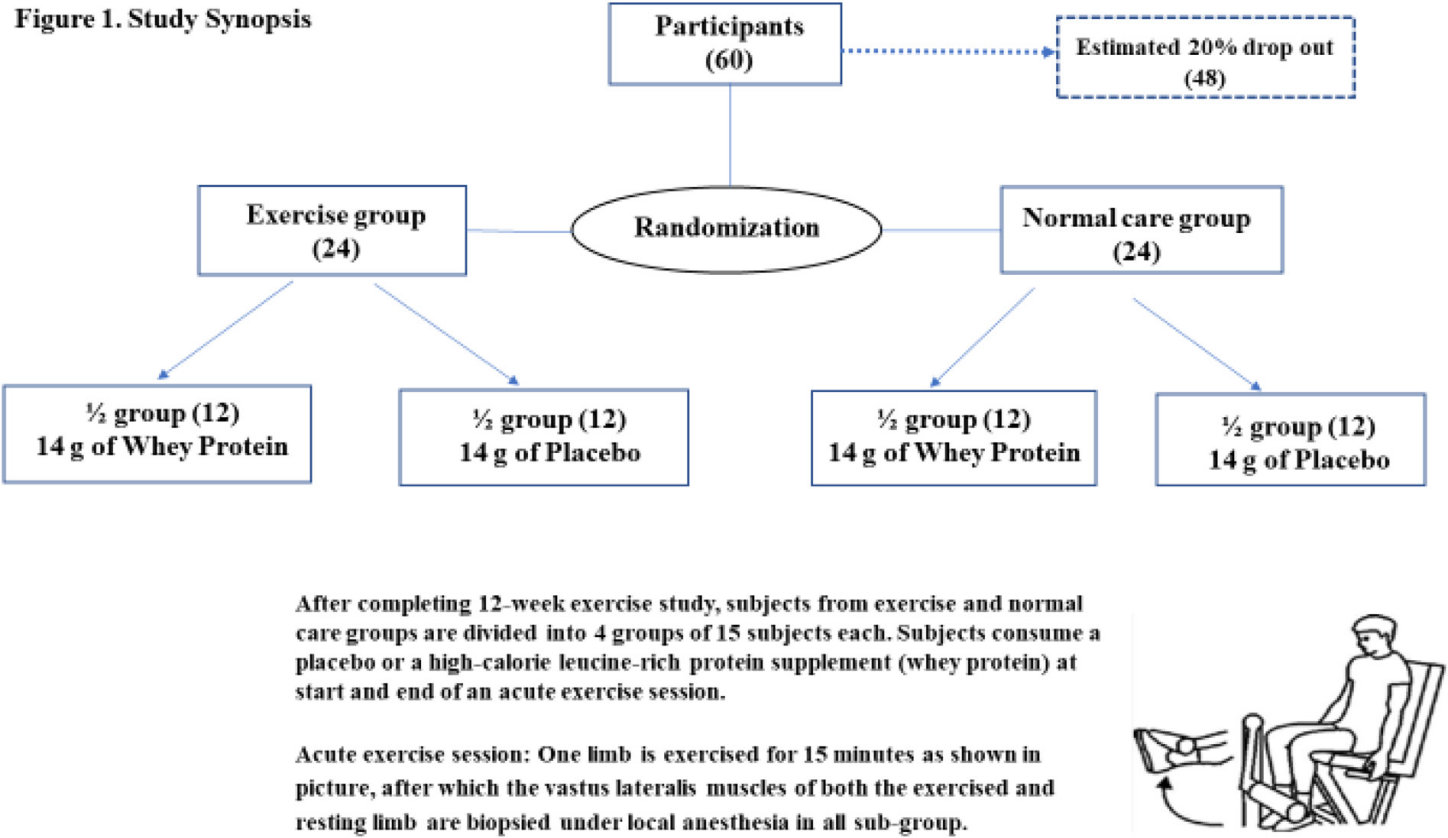
Study synopsis.

A design synopsis of the study is presented in Fig. 1. One group will undergo a home-based exercise program and the other normal care over a 12-week period. In order to assess the effects of acute and chronic exercise and protein supplementation on protein signaling, at the end of 12 weeks half the participants in each group will be randomly assigned to receive a blinded nutritional supplement (high-calorie whey or placebo) at the start and end of a bout of acute unilateral lower limb exercise, followed by a muscle biopsy. The blind will be broken upon completion of the study.

***Statistical Analysis Plan.*** Comparisons of clinical and demographic data between groups at baseline are performed by the appropriate statistical test (for continuous variables, *t*-test for normally distributed data and Wilcoxon test for non-normal data; for categorical variables, chi-square test or Fisher's exact test). These comparisons are performed at baseline only to assess adequacy of randomization. Comparisons between groups for cardiopulmonary exercise test, muscle biopsy, energy expenditure, cognitive function, and other functional measures before and after the study period are performed by multivariate ANOVA; post-hoc comparisons are made using the Bonferroni method. To explore the relationships between a given outcome and other variables, multiple regression techniques are used. For the primary outcome, change in peak VO_2_, the specific independent variables in the model include exercise compliance (average energy expended in kcals/week), change in muscle strength, and change in muscle mass. Missing data are managed using the multiple imputation method described by Schafer & Olsden [16].

## 5 Preliminary results

**Fig. 2:**
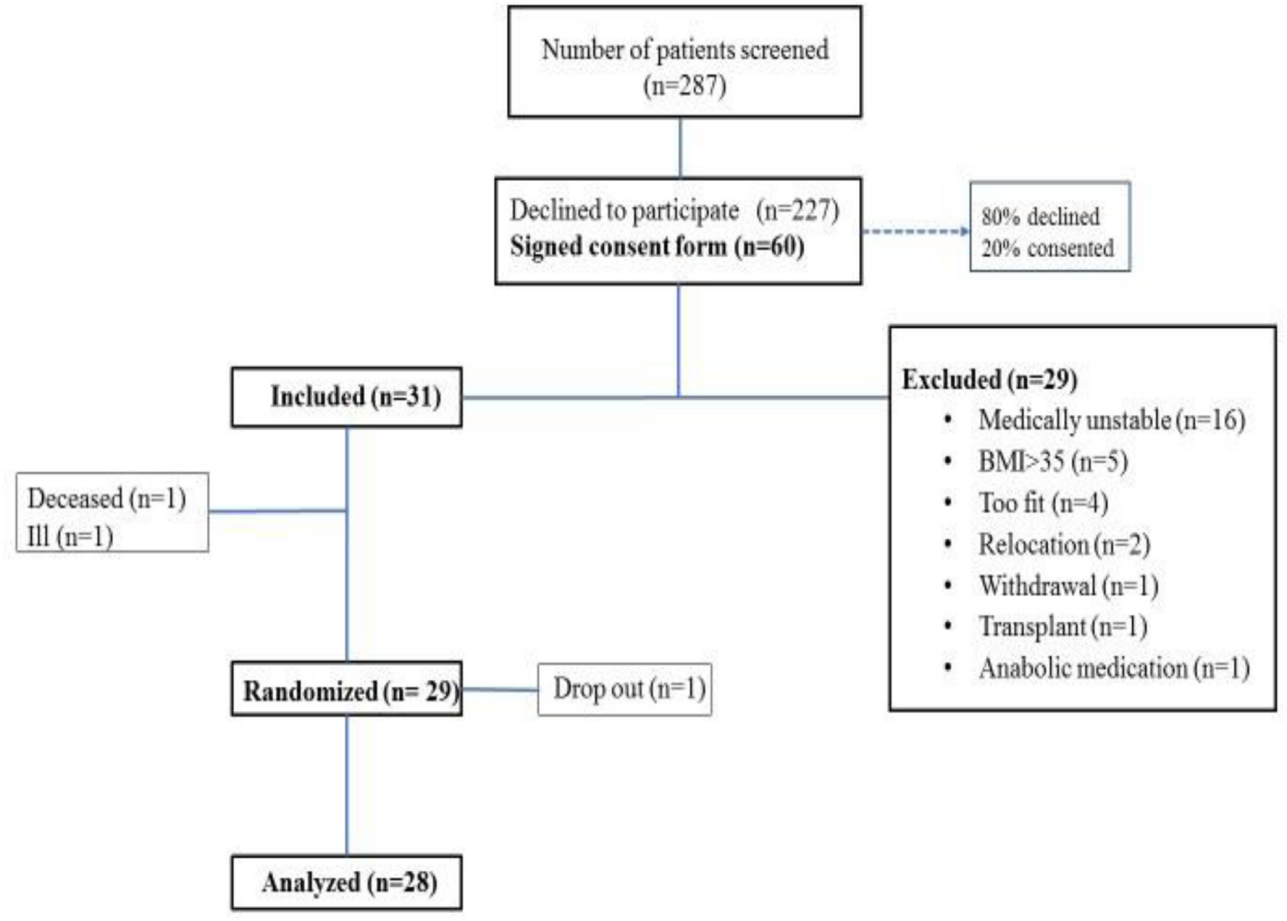
Recruitment flow chart.

***Recruitment and enrollment.*** Fig. 2 summarizes the flow of potential participants from recruitment to randomization. We approached 287 potential subjects from local Satellite Dialysis clinics, the Palo Alto VA Dialysis Center and the Stanford *TRAC* clinic. Two hundred twenty-seven patients (80%) declined to participate. Sixty patients signed consent forms. Among the 60 who were interested in participating, 29 (48%) were excluded; most for medical instability after enrollment, although some were excluded due to language barriers. The remaining 31 (52% of interested participants) qualified for the study. Of these, one died and one became very ill for reasons unrelated to the study. We randomized the remaining 29 participants. There was one dropout. For this preliminary analysis of baseline parameters, 28 participants are included.

***Baseline characteristics.*** Table 2 provides demographic and clinical characteristics of the participants. The average BMI was 28 kg/m^2^. Seventy percent of participants had Type II diabetes and all subjects had a history of hypertension. Table 3 describes baseline measures for exercise capacity, functional tests, strength, body composition, and cognitive and health status. From these baseline measures, mean peak VO_2_ (14.7 ± 3.3 ml/kg/min) represented 55% of the age-predicted value (using the Fitness Registry and the Importance of Exercise: A National Data Base [FRIEND] registry [17].Mean values for percent predicted 6-min walk and 60 s sit-to-stand test were 69% and 55%, respectively. Upper and lower body strength were also low relative to age-related norms. Total body fat % determined by DXA was 36 ± 7, representing 133 ± 22% of the age-predicted value. From the health status survey, physical and mental component scores as well as quality of life scores were lower than reference values. Similarly, the kidney disease-specific quality of life assessment showed that participants were negatively influenced by their condition.

**Table 2:** Demographic and clinical characteristics of participants.

	EX *(n* = *13)*	NC *(n* = *15)*	Total *(n* = *28)*
**Demographic**			
Age (years)	66.5 ± 7.4	66.3 ± 6.7	66.4 ± 6.9
Gender			
Male	11	10	21
Female	2	5	7
Race (%)			
Asian	7 (53.9%)	2 (13.3%)	9 (32.1%)
Hispanic	1 (7.7%)	5 (33.3%)	6 (21.4%)
Caucasian	1 (7.7%)	5 (33.3%)	6 (21.4%)
Pacific Islander	3 (23%)	1 (6.7%)	4 (14.3%)
African American	1 (7.7%)	2 (13.3%)	3 (10.7%)
**Anthropomorphic**			
BMI (kg/m2)	28.5 ± 3.3	29.1 ± 4.6	28.8 ± 4.0
Waist/Hip ratio	1.0 ± 0.1	1.0 ± 0.1	1.0 ± 0.1
Mid-Arm Circumference	12.1 ± 1.7	11.6 ± 1.4	11.9 ± 1.6
**Adiposity**			
Skin Fold Thickness (inches)			
Abdominal	17.7 ± 7.3	18.8 ± 6.7	18.3 ± 6.9
Suprailiac	13.0 ± 8.1	12.6 ± 4.5	12.8 ± 6.3
Tricep	13.0 ± 5.4	14.5 ± 5.8	13.8 ± 5.5
**Medical History**			
Hypertension	13 (100%)	15 (100%)	28 (100%)
Type II DM	9 (69%)	10 (66.7%)	19 (67.9%)
CAD	5 (38.5%)	5 (33.3%)	10 (35.7%)
Stroke	3 (23%)	3 (20%)	6 (21.4%)
Liver Disease	1(7.7%)	3 (20%)	4 (14.3%)
Cancer	1(7.7%)	3 (20%)	4 (14.3%)

**Table 3:** Baseline measurements of exercise and functional capacity, strength, body composition, health status and cognitive tests.

	EX (*n* = *13*)	NC (*n* = *15*)	Total (*n* = *28*)
**Exercise and functional capacity**			
Max VO2 (ml/kg/min)	14.5 ± 3.3	15.1 ± 3.3	14.7 ± 3.3
% predicted value of peak VO2	51 ± 13	59 ± 15	55 ± 14
Chair Raise Test (# of repetition)	19 ± 9	18 ± 8	18 ± 8.2
% predicted value of chair rise test	55 ± 26	56 ± 18	55 ± 22
6MWT (meter)	333 ± 80	313 ± 65	322 ± 71
% predicted value of 6MWT	72 ± 16	66 ± 13	69 ± 16
**Strength**			
Hand grip	54 ± 15	53 ± 16	54 ± 16
% predicted value of hand grip	68 ± 19	68 ± 16	68 ± 16
Upper body strength (lbs.)	69 ± 28	59 ± 31	64 ± 29
Lower body strength (lbs.)	71 ± 23	70 ± 26	71 ± 24
**Body composition**			
Total leg mass (kg)	23 ± 4	24 ± 4	23 ± 4
Total body mass (kg)	79 ± 15	81 ± 14	80 ± 14
Total body fat (%)	35 ± 7	37 ± 7	36 ± 7
% predicted value of body fat	133 ± 25	133 ± 18	133 ± 22
**Health status**			
SF-36 PCS	39 ± 11	43 ± 7	41 ± 9
SF-36 MCS	49 ± 14	54 ± 10	52 ± 12
KDQOL-Burden	44 ± 33	49 ± 31	47 ± 31
KDQOL- Effect	65 ± 25	76 ± 20	71 ± 23
**Cognitive (Beeson Cognitive Test)**			
Rey Auditory verbal learning words	31 ± 8	35 ± 9	33 ± 9
Trails A	60 ± 26	61 ± 35	61 ± 30
Trails B	185 ± 91	171 ± 75	178 ± 81
Digital symbol	40 ± 17	41 ± 15	40 ± 16
COWAT	25 ± 15	31 ± 10	28 ± 13

EX = exercise group, NC = normal care group, KDQOL= Kidney disease quality of life, CRF = chronic renal failure, COWAT = controlled-oral word association, PCS = physical component score, MCS = mental component score.

## 6 Discussion

MHD patients tend to lose muscle mass in part related to the fact that uremia contributes to fatigue and therefore a lack of motivation to exercise; these factors are exacerbated after receiving dialysis treatment which itself contributes to fatigue and further limits daily activities. Most dialysis patients receive dialysis treatment in a clinic three or four times per week, and average dialysis time is three or 4 h. Thus, time is another important barrier to exercise for many dialysis patients. Moreover, programs to encourage physical activity for these patients are lacking, and thus there tends to be a high rate of disuse-related disability. The current trial is a comprehensive investigation of the potential benefits of home-based exercise in terms of improving exercise capacity and preventing loss of muscle mass and function in elderly MHD patients. The “hybrid” nature of exercise program (home-based following several in-person sessions) is important given that transportation barriers are removed which enhances compliance. Close case management also provides support which further improves compliance, allows the provider to better quantify adherence to physical activity and provides surveillance for any health issues that inevitably arise with patients who have numerous co-morbidities such those with CKD. Data obtained from muscle biopsies may provide novel insights into the mechanisms whereby resistance exercise induces muscle enlargement and whether the anabolic response to exercise can be enhanced by providing a leucine-rich protein supplement at the time of exercise. The current study has the potential to provide both clinicians and patients an impetus to incorporate physical activity into the daily lives of MHD patients, in addition to informing the Nephrology community regarding mechanisms by which regular exercise may enhance muscle growth and function.

The evaluation of protein signaling is a novel feature of the study. There are several reasons why we suspect exercise will enhance protein signaling. In CKD, there is a change in the balance between local IGF-1, a major regulator of muscle growth and repair, and myostatin, an inhibitor of muscle growth. Local IGF1 is decreased and myostatin is increased with regular exercise [18,19]. In addition to IGF, the Akt/mTOR pathway plays an important role in promoting muscle growth and repair [[20], [21], [22], [23], [24]]. Exercise is known to induce mechanical stimuli that activate the Akt/mTOR pathway [25,26]. Notably, mTOR signaling is directly activated by essential amino acids (EAA), particularly leucine. mTOR, via its downstream signaling proteins, then up-regulates mRNA translation initiation and thereby protein synthesis [11,27]. In non-CKD subjects, leucine-rich supplements are highly effective in promoting muscle protein synthesis especially when combined with resistance exercise [25,28,29]. A similar response is seen with endurance exercise [29]. Whey is of special interest among commonly used protein supplements because it is readily digestible, inexpensive, and rich in EAA (particularly leucine). It also has low sodium, potassium, and phosphorus contents suitable for patients with ESRD [25,30,31]. We anticipate that by quantifying the Akt/mTOR signaling pathway in the skeletal muscle after acute and chronic exercise, novel insights will be gained regarding the mechanisms of muscular adaptations to exercise in patients with CKD.

Through case-management, we anticipate that most patients will be motivated to become more active in their daily lives with the program. Data from patients with other chronic conditions suggest that a better understanding of a given patients’ medical, social and psychological conditions and close monitoring positively influence QOL and the ability to become more physically active. Baseline findings using a CKD-specific QOL instrument suggest that most patients (67%) felt that they are a burden to society due to their illness and thus prefer not to engage in social activities. Along with perceived safety risks, this undoubtedly further contributes to poor adherence to exercise. Despite these barriers, a growing body of evidence suggests that functional performance improves following exercise training in patients with CKD. The potential benefits of this trial extend beyond improving physical function to increasing confidence in exercise as well as activities of daily living.

### 6.1 Limitations of the study

Recruitment is challenging due to the muscle biopsy component of the study, and the reluctance of patients to undergo an invasive procedure. Another barrier has been patient availability due to the time-consuming nature of dialysis days. Several participants were excluded for having peak VO_2_ measurements that were out of the study range (either too high or too low). Finally, this population suffers from numerous co-morbidities that limit both their ability and motivation to participate in an exercise program.

### 6.2 Summary

Most of the available studies on exercise therapy in patients with MHD have involved small numbers, lack randomized controls, were of short duration, and enrolled comparatively young subjects in relatively good condition. There is a dearth of studies specifically targeting the elderly. Despite its numerous documented benefits, implementation of rehabilitation as a standard treatment modality in patients with CKD is lacking. Not surprisingly, virtually all of the baseline functional measures from our preliminary results were markedly impaired relative to those for age-matched normal standards. Therefore, MHD patients have a considerable amount to gain from participation in an exercise program. There is a need for large-scale prolonged studies to establish the true long-term benefits of exercise on morbidity and mortality in this population, but a prerequisite to such studies is the development of a low-cost exercise program that will be relatively easy to administer and adhere to, and that will form part of routine MHD care. It may be that given the natural limit of the lifespan and the co-morbid conditions common in elderly MHD patients, the main benefits of exercise are improvements in physical function, activities of daily living, QOL, and a decrease in adverse events (e.g. falls and hospitalizations, which are more frequent in the elderly dialysis patient than the general population). If the program described herein were to be implemented on a wider scale, the overall health of patients with advanced CKD requiring MHD could potentially be markedly improved, and healthcare cost savings could be substantial. Moreover, if effective, the study may serve as a model for implementing home-based exercise for advanced CKD patients not needing MHD and for establishing clinic-based exercise programs for MHD patients unable to undergo home exercise. Finally, this program could serve as a model for the management of patients with other chronic disease states such as advanced COPD.
